# Residence and young women’s comprehensive HIV knowledge in Ethiopia

**DOI:** 10.1186/s12889-020-09687-1

**Published:** 2020-10-23

**Authors:** Biruk Beletew Abate, Ayelign Mengesha Kassie, Melese Abate Reta, Gillian H. Ice, Zelalem T. Haile

**Affiliations:** 1 Department of Nursing, College of Health Sciences Woldia University, P.O.Box 400, Woldia, Ethiopia; 2 Department of Medical Microbiology, Faculty of Health Sciences, University of Pretoria, Pretoria, South Africa; 3 Department of Medical Laboratory Science, College of Health Sciences, Woldia University, Woldia, Ethiopia; 4 Department of Social Medicine, Ohio University Heritage College of Osteopathic Medicine, Athens, OH USA; 5 Department of Social Medicine, Ohio University Heritage College of Osteopathic Medicine, Dublin, OH USA

**Keywords:** Comprehensive HIV knowledge, Young women, Residence, Demographic and health survey, Ethiopia

## Abstract

**Background:**

Human immunodeficiency virus (HIV) infection is a global health problem. The epidemic is very serious in sub-Saharan Africa with approximately 70% of the global cases. The disease particularly affects youth, accounting for half of the new HIV infections yearly. Inadequate knowledge may contribute to the high rates among youth. Hence, the main aim of this study was to examine the association between residence and comprehensive HIV knowledge among women aged 15–24 years in Ethiopia.

**Methods:**

This cross-sectional study used nationally representative data from the 2016 Ethiopian demographic health survey (*n* = 5926). Chi-square tests and multivariable logistic regression modeling were performed.

**Results:**

Approximately 23.9% of the study participants had a comprehensive HIV knowledge and 74.7% were rural residents. In the multivariable-adjusted model, we found a significant interaction between place of residence and HIV testing on comprehensive HIV knowledge (*P* for interaction = 0.005). In the subgroup analysis, a statistically significant associations between place of residence and comprehensive HIV knowledge was found only in women who have never been tested for HIV. In this subgroup, rural women had lower odds of having a comprehensive HIV knowledge compared to their urban counterparts (OR 0.42, 95% CI: 0.23–0.74; *P = 0.003*). Furthermore, in the subgroup of women who have never been tested for HIV, education and region were significantly associated with comprehensive HIV knowledge. Compared to women with no education, the odds of having a comprehensive HIV knowledge were higher in women who had primary (OR 2.86, 95% CI: 1.63–5.02; *P < 0.001*) and secondary or above education (OR 5.49, 95% CI: 2.92–10.32; *P < 0.001*), respectively. The odds of having a comprehensive HIV knowledge were lower in women from the Somali region compared to women from Addis Ababa region (OR 0.41, 95% CI: 0.18–0.90; *P = 0.027*).

**Conclusions:**

Rural residence was negatively associated with comprehensive HIV knowledge only in women who have never been tested for HIV. These findings suggest that the development and implementation HIV education and awareness programs should target rural areas, especially where there is limited access to HIV testing.

## Background

Globally, HIV is the leading cause of death among women of the reproductive age group, and unprotected sex is the main driver of HIV transmission [1, 2]. Youth, aged 15–24, are the key affected among women and account for more than 30% of the new infections reported in 2017 by UNICEF [3]. Approximately 70% of all people living with HIV live in Sub-Saharan Africa [4]. According to World Health Organization (WHO) report, in 2012, about 2.1 million (5.9%) of the total population living with HIV were adolescents and several risky sexual behaviors such as early sexual initiation (before 18 years old), having multiple sexual partners, unprotected sexual intercourse, having a high-risk partner and engaging in sex work were identified as very important factors putting adolescents into a higher risk for acquiring HIV [5–8].

Adolescence is a critical stage of life where adolescents attain increased capacity for complex problem-solving and critical thinking skills. However, this maturation process coincides with increased risk-taking as well as increased significance of peer influences on the adolescents [9]. Yet, this age group still lacks access to sexual and reproductive health programs that provide essential information, services and social support to prevent and care for HIV and other sexually transmitted disease, especially in low- and middle income countries [9].

In their transition to adulthood, adolescents experience many economic and social pressures such as becoming sexually active as well as drug and alcohol use. This makes them more susceptible to sexual coercion and abuse and puts them at a higher risk of HIV acquisition [10]. Globally, less than 30% of young women have comprehensive HIV/AIDS knowledge. Lack of accurate and complete HIV knowledge is one of the contributing factors that increases the number of new HIV infections among young people worldwide. Therefore, increasing individual’s knowledge level and behavioral change remains the world’s primary tool for achieving HIV prevention goal [11]. Sub-Saharan Africa remains among the toughest hit regions by the pandemic, with nearly one in every 25 adults (4.2%) living with HIV, accounting for nearly two-thirds of the global total HIV cases [12, 13]. In 2018, approximately 79% of perintally infected adolescents’, adolescents were living in sub-Saharan Africa [14].

Worldwide, about 74.9 million people became infected with HIV in 2018 [1]. In sub-Saharan Africa, while much progress has been made on HIV prevention and treatment, incidence rates are increasing among youth between 10 and 19 years. Youth from sub-Saharan Africa account for 73.8% of new infections among youth aged 15–19.Worldwide, one third of new HIV infections occur among youths (42%), of which, 73% living in Sub-Saharan Africa and only one in five adolescent girls in sub-Saharan Africa are aware of their HIV status. The increasing rates among youth led many advocates to call for a refocusing ofn prevention to this neglected age group [9]. Southern Africa is the worst affected region on the continent [15]. Half of the population in Uganda had comprehensive knowledge of HIV prevention [16]. Significantly low levels of comprehensive HIV knowledge (9.4%) were observed in Nigeria [17].

Ethiopia is among the countries that are heavily affected by HIV, and the disease is one of the top ten causes of death in the country [10]. In Ethiopia, over the past two decades HIV prevalence rate decreased from 3.3% in 2000 to 0.9% in 2017, and AIDS-related deaths from 83,000 deaths in 2000 to 15,600 in 2017 [13]. In 2017, there were 720,000 people living with HIV and 27,104 newly diagnosed cases [18]. According to USAID report in Ethiopia, 690,000 people were living with HIV in 2018; 65% of people living with HIV were on ART in the same year [19]. AIDS estimated deaths in Ethiopia were reported at 11000 in 2018 [20]. In Ethiopia, only about a quarter of adolescents had comprehensive HIV knowledge [21]. Urban-rural residence has been identified as an important predictor of HIV/AIDS knowledge. Some recent studies [22–24] have demonstrated that adolescents living in rural areas are less likely to have comprehensive knowledge about HIV and AIDS. Existing literature examining the relationship between residence and comprehensive HIV knowledge among young women in Ethiopia is limited. Ascertaining these variations could be essential in identifying target areas for HIV/AIDS education programs. Therefore, the aim of this study was to assess the relationship between residence and comprehensive knowledge of HIV among young women aged 15–24 years in Ethiopia.

## Methods

### Study design and collected data

The current study is based on the 2016 Ethiopian Demographic and Health Survey (EDHS). The 2016 EDHS is the fourth survey conducted in the country. This survey collected information on household’s and respondent’s characteristics, infants and child mortality, malaria, maternal health, maternal mortality, nutrition, tobacco use, wealth, women’s empowerment, anemia child health, domestic violence, educational status, environmental health, family planning, female genital mutilation, fertility and fertility preferences, gender/domestic violence and HIV knowledge. The primary objective of the 2016 EDHS was to provide up-to-date estimates of key demographic and health indicators of the Ethiopian population [25]. The 2016 EDHS included a nationally representative sample of women (aged 15–49 years) and men (aged 15–59 years) from the nine regions and two administrative cities in the country [25, 26].

### Sampling techniques and study population

A stratified two stage sampling method was used to produce a representative sample for the country as whole. Each region was stratified into urban and rural areas, which yielded 21 sampling strata. Samples of enumeration areas (EAs) were selected independently in each stratum in two stages. Implicit stratification and proportional allocation were achieved at each of the lower administrative levels by sorting the sampling frame within each sampling stratum before sample selection according to administrative units in different levels, and by using a probability proportion to size selection at the first stage of sampling. In the first stage, 645 EAs were selected with probability proportional to the EA size and with independent selection in each sampling stratum. The EA size is the number of residential households in the EA as determined in the 2007 Ethiopian Population and Housing Census (EPHC).

A household listing operation was implemented in the selected EAs, and the resulting lists of households served as the sampling frame for the selection of households in the second stage. To minimize the task of household listing, the selected large EAs with more than 200 households were segmented. Only one segment was selected for the survey with probability proportional to the segment size. Household listing was conducted only in the selected segment. Thus, a 2016 EDHS cluster is either an EA or a segment of an EA. In the second stage of selection, a fixed number of 28 households per cluster were selected with an equal probability systematic selection from the newly created household listing. Only members of pre-selected households were interviewed. No replacements or changes of the pre-selected households were allowed in the implementing stages to prevent bias. All women aged 15–49 years who were usual members of the selected households or who spent the night before the survey in the selected households were eligible for the female survey [26]. For the current study, the sampling frame consists of young women aged 15–24 years (*n* = 6401). We excluded women with missing data on the variables of interest for the current study (*n* = 475). The final study sample consisted of 5926 women.

### Data collection tool and procedure

Data collection for this survey took place from January 18 to June 27, 2016. Five questionnaires were used for the 2016 EDHS: The Household Questionnaire, the Woman’s Questionnaire, the Man’s Questionnaire, the Biomarker Questionnaire, and the Health Facility Questionnaire. These questionnaires were adapted from the DHS Program’s standard Demographic and Health Survey questionnaires in a way to reflect the population and health issues relevant to Ethiopia [27]. For the purpose of this study, data on women captured in the 2016 EDHS was used.

### Variables

The outcome variable was comprehensive knowledge on HIV among young women. Each woman was asked whether or not she agreed or disagreed with the following five items regarding transmission and misconceptions on HIV/AIDS: 1) HIV can be prevented by using condoms every time you have sexual intercourse; 2) HIV can be prevented by limiting intercourse to one uninfected partner; 3) it is possible for a healthy-looking person to have the AIDS virus; 4) the AIDS virus can be transmitted from mosquito bites; 5) a person can get HIV by sharing food with a person who has AIDS [28]. First an additive summary correct response was created to derive a comprehensive knowledge on HIV score. This score is then dichotomized resulting in a binary variable with 0 (at least one incorrect response) and 1 (correct response to the 5 questions).

The primary exposure of interest was residence which was defined as urban and rural. Covariates for the study were selected based on of existing literature and included respondent’s age (“15–19”, “20–24”), marital status (“never married”, “currently married/living together”, “divorced/separated/widowed”), highest level of education (“no education”, “primary”, “secondary or higher”), household wealth index (“poorest”, “poorer”, “middle”, richer”, “richest”), religion (“Christian”, “other”), region (nine geographical regions and two administrative cities), ever been tested for HIV (“no”, “yes”), mass media exposure (“no”, “yes”), internet access (“no”, “yes”), distance to a health facility (“not a big problem”, “a big problem”). Household wealth index is a composite measure of a household’s living standard categorized into 5 quintiles which was calculated using household’s ownership of selected assets, such as televisions and bicycles, materials used for housing construction, types of water access and sanitation facilities. Mass media exposure was measured based on how often respondents read a newspaper, listened to the radio, or watched television. Those who responded at least once a week to one of these sources are considered regularly exposed to that form of media***.***

### Statistical analysis

Descriptive statistics were performed to provide a summary of the characteristics of the study sample. Rao-Scott adjusted chi-square statistic test statistic was used to examine differences in the proportions of having comprehensive HIV knowledge by place of residence and each of the covariates. Multiple logistic regression models were then used to determine the independent association of place of residence and comprehensive HIV knowledge while adjusting for the covariates. To control for confounding each covariate was retained in the multivariable-adjusted model regardless of statistical significance in the bivariate analysis. Pairwise interactions between place of residence and each covariate on comprehensive HIV knowledge were assessed. To assess the presence of collinearity, we employed regression diagnostics using a conservative cut-off value for variance inflation factor > 4. Using this approach, we found no significant collinearity between variables included in the multivariable model. The corresponding odds ratio and 95% confidence interval for the association between the place of residence and comprehensive HIV knowledge stratified by type of were reported. Two-tailed *p*-value < 0.05 was considered statistically significant. Sampling weights that accounted for complex survey design were incorporated in all analyses. All statistical analyses were conducted using SPSS for Windows (version 24; IBM Corp).

## Results

Table 1 presents the characteristics of the study sample. Overall, a greater proportion of the participants were younger, never been married, had primary education and were from relatively rich households. Most of the participants were not exposed mass media and the internet, have never been tested for HIV and reported that distance to a health facility is a not a big problem. Approximately 23.9% of the study participants had a comprehensive HIV/AIDS knowledge and 74.7% were rural residents. Participants from rural areas were more likely to be married, reside in poor households, were less likely to have access to media and the internet and were less likely to have been tested for HIV (Table 1).

**Table 1:** Descriptive statistics of the study sample (*n* = 5926)

		Place of residence		
	Overall n (wt.%)	Urban n (wt.%)	Rural n (wt.%)	*p*
**Age**				0.071
15–19	3212 (54.3)	1216 (54.7)	1996 (54.2)	
20–24	2714 (45.7)	1080 (45.3)	1634 (45.8)	
**Marital status**				
Never married	3382 (57.5)	1646 (74.2)	1736 (51.9)	< 0.001
Married/Living together	2236 (36.9)	548 (22.0)	1688 (42.0)	
Divorced/separated/widowed	308 (5.5)	102 (3.8)	206 (6.1)	
**Education**				< 0.001
None	1103 (17.3)	142 (3.7)	961 (21.8)	
Primary	2948 (55.4)	932 (36.4)	2016 (61.8)	
Secondary or above	1875 (27.3)	1222 (60.0)	653 (16.4)	
**Household wealth index**				< 0.001
Poorest	1181 (13.9)	30 (1.7)	1151 (18.0)	
Poorer	714 (17.2)	19 (0.9)	695 (22.7)	
Middle	748 (18.0)	21 (0.6)	727 (23.8)	
Richer	800 (20.5)	41 (1.8)	759 (26.8)	
Richest	2483 (30.5)	2818 (94.9)	298 (8.7)	
**Religion**				< 0.001
Christian	3668 (70.3)	1588 (81.7)	2080 (66.5)	
Other	2258 (29.7)	708 (18.3)	1550 (33.5)	
**Region**				< 0.001
Addis Ababa	793 (7.0)	793 (27.7)	0 (0.0)	
Tigray	726 (8.5)	196 (8.8)	530 (8.4)	
Afar	452 (0.9)	84 (1.0)	368 (0.9)	
Amhara	611 (23.3)	88 (17.6)	523 (25.3)	
Oromia	662 (34.7)	104 (25.1)	558 (38.0)	
Somali	399 (2.2)	115 (2.2)	284 (2.3)	
Beniishangul	421 (1.1)	77 (1.0)	344 (1.1)	
SNNPR	689 (21.1)	106 (13.5)	583 (23.6)	
Gambella	371 (0.3)	158 (0.6)	213 (0.2)	
Harari	360 (0.3)	214 (0.6)	146 (0.1)	
Dire Dawa	442 (0.6)	361 (1.8)	81 (0.2)	
**Ever tested for HIV**				< 0.001
No	3266 (59.7)	975 (44.5)	2291 (64.9)	
Yes	2660 (40.3)	1321 (55.5)	1339 (35.1)	
**Mass media exposure**				< 0.001
No	3583 (68.1)	649 (29.5)	2934 (81.1)	
Yes	2343 (31.9)	1647 (70.5)	696 (18.8)	
**Internet access**				< 0.001
No	5132 (91.9)	1604 (74.4)	3528 (97.9)	
Yes	794 (8.1)	692 (25.6)	102 (2.1)	
**Distance to health facility**				< 0.001
Not a big problem	2386 (46.9)	447 (17.6)	1939 (56.8)	
A big problem	3540 (53.1)	1849 (82.4)	1691 (43.2)	
**Comprehensive HIV knowledge**				< 0.001
No	4541 (76.1)	1513 (59.1)	3028 (81.8)	
Yes	1385 (23.9)	783 (40.9)	602 (18.2)	

Abbreviations: *Wt.%* Weighted percent; *SNNPR* Southern Nations, Nationalities, and Peoples’ Region

The bivariate distributions of comprehensive HIV knowledge are shown in Table 2. Participants who had a comprehensive HIV knowledge were more likely to be never been married, have secondary or above education, live in relatively rich households, have exposure to mass media and the internet, have been tested for HIV previously, and reported that distance to a health facility is a not a big problem. Compared to rural residents, a significantly higher proportion of participants who reside in urban areas had comprehensive HIV knowledge (40.9% vs 18.2%, *p* < 0.001) (Table 2). In the multivariable-adjusted model, we found a significant interaction between place of residence and HIV testing on comprehensive HIV knowledge (*P* for interaction = 0.005). The stratified analysis revealed place of residence was significantly associated with comprehensive HIV/AIDS knowledge, but only in women who have never been tested for HIV. In this subgroup, rural women had lower odds of having a comprehensive HIV knowledge compared to their urban counterparts (OR 0.42, 95% CI: 0.23–0.74; *P = 0.003*). Furthermore, in the subgroup of women who have never been tested for HIV, education and region were significantly associated with comprehensive HIV knowledge. Compared to women with no education, the odds of having a comprehensive HIV knowledge were higher in women who had primary (OR 2.86, 95% CI:1.63–5.02; *P < 0.001*) and secondary or above education (OR 5.49, 95% CI: 2.92–10.32; *P < 0.001*), respectively. The odds of having a comprehensive HIV knowledge were lower in women from the Somali region compared to women from Addis Ababa region (OR 0.41, 95% CI: 0.18–0.90; *P = 0.027*) (Table 3).

**Table 2:** Characteristics of the study sample by comprehensive HIV knowledge (*n* = 5926)

	Comprehensive HIV knowledge		
	No n (wt.%)	Yes n (wt.%)	*P*
**Age**			0.831
15–19	2472 (75.9)	740 (24.1)	
20–24	2069 (76.3))	645 (23.7)	
**Marital status**			
Never married	2456 (71.8)	926 (28.2)	< 0.001
Married/living together	1847 (82.3)	389 (17.7)	
Divorced/separated/widowed	238 79.3)	70 (20.7)	
**Education**			< 0.001
None	1028 (92.5))	75 (7.5)	
Primary	2354 (80.0)	594 (20.0)	
Secondary or above	1159 (57.9)	716 (42.1)	
**Household wealth index**			< 0.001
Poorest	1069 (88.2)	112 (11.8)	
Poorer	607 (84.3)	107 (15.7)	
Middle	616 (82.5)	132 (17.5)	
Richer	607 (77.2)	193 (22.8)	
Richest	1642 (61.4)	841 (38.6)	
**Religion**			< 0.001
Christian	2627 (72.9)	1041 (27.1)	
Other	1914 (83.7)	344 (16.3)	
**Region**			< 0.001
Addis Ababa	496 (62.2)	297 (37.8)	
Tigray	506 (70.5)	220 (29.5)	
Afar	397 (86.8)	55 (13.2)	
Amhara	440 71.0)	171 (29.0)	
Oromia	534 (80.1)	128 (19.9)	
Somali	367 (93.0)	32 (7.0)	
Beniishangul	345 (80.6)	76 (19.4)	
SNNPR	537 (79.3)	152 (20.7)	
Gambella	279 (73.7)	92 (26.3)	
Harari	286 (79.0)	74 (21.0)	
Dire Dawa	354 (80.7)	88 (19.3)	
**Ever tested for HIV**			< 0.001
No	2662 (80.7)	604 (19.3)	
Yes	1879 (69.2)	781 (30.8)	
**Mass media exposure**			< 0.001
No	2971 (81.4)	612 (18.6)	
Yes	1570 (64.8)	773 (35.2)	
**Internet access**			< 0.001
No	4095 (78.3)	446 (21.7)	
Yes	1037 (51.1)	348 (48.9)	
**Distance to health facility**			< 0.001
Not a big problem	2010 (83.4)	376 (16.6)	
A big problem	2531 (69.6)	1009 (30.4)	
**Place of residence**			< 0.001
Urban	1513 (59.1)	783 (40.9)	
Rural	3028 (81.8)	602 (18.2)	

Abbreviations: *Wt.%* Weighted percent; *SNNPR* Southern Nations, Nationalities, and Peoples’ Region

Row percentages are presented

**Table 3:** Association between place of residence and comprehensive HIV knowledge stratified by ever tested for HIV (*n* = 5926)

	Ever tested for HIV			
	No (*n* = 3266)		Yes (*n* = 2660)	
	AOR (95% CI)	*p*	AOR (95% CI)	*P*
**Age**				
15–19	Reference		Reference	
20–24	0.95 (0.66, 1.35)	0.796	1.20 (0.91, 1.59)	0.191
**Marital status**				
Never married	Reference		Reference	
Married/livingtogether	0.89 (0.56, 1.41)	0.591	0.62 (0.46, 0.83)	0.002
Divorced/separated/widowed	0.59 (0.27, 1.28)	0.165	0.87 (0.49, 1.52)	0.615
**Education**				
None	Reference		Reference	
Primary	2.86 (1.63, 5.02)	< 0.001	1.87 (1.07, 3.27)	0.029
Secondary or above	5.49 (2.92, 10.32)	< 0.001	2.87 (1.62, 5.07)	< 0.001
**Household wealth index**				
Poorest	Reference		Reference	
Poorer	1.91 (0.70, 2.02)	0.517	0.93 (0.52, 1.67)	0.799
Middle	1.60 (0.98, 2.59)	0.058	0.59 (0.33, 1.06)	0.078
Richer	1.61 (1.00, 2.59)	0.050	0.83 (0.48,1.43)	0.508
Richest	1.39 (0.71, 2.73)	0.334	1.29 (0.64, 2.23)	0.568
**Religion**				
Christian	Reference		Reference	
Other	0.77 (0.50, 1.18)	0.224	1.05 (0.71, 1.55)	0.780
**Region**				
Addis Ababa	Reference		Reference	
Tigray	1.43 (0.82, 2.46)	0.206	1.65 (1.06, 2.55)	0.026
Afar	1.26 (0.61, 2.60)	0.530	0.92 (0.57, 1.48)	0.729
Amhara	1.65 (0.90, 3.02)	0.104	1.95 (1.23, 3.08)	0.004
Oromia	1.26 (0.74, 2.15)	0.388	1.24 (0.70, 2.21)	0.457
Somali	0.41 (0.18, 0.90)	0.027	0.72 (0.29, 1.79)	0.483
Beniishangul	0.80 (0.41, 1.55)	0.504	1.19 (0.65, 2.17)	0.568
SNNPR	0.96 (0.56, 2.31)	0.876	1.46 (0.93, 2.31)	0.099
Gambella	1.14 (0.56, 2.31)	0.714	1.02 (0.66, 1.59)	0.920
Harari	0.57 (0.28, 1.14)	0.112	0.90 (0.48, 1.67)	0.733
Dire Dawa	0.64 (0.36, 1.15)	0.136	0.57 (0.34, 0.95)	0.033
**Mass media exposure**				
No	Reference		Reference	
Yes	1.18 (0.80, 1.74)	0.416	1.22 (0.90, 1.66)	0.204
**Internet access**				
No	Reference		Reference	
Yes	1.08 (0.67,1.73)	0.757	1.50 (1.01, 2.22)	0.050
**Distance to health facility**				
Not a big problem	Reference		Reference	
A big problem	1.20 (0.90, 1.61)	0.209	1.33 (0.95, 1.86)	0.101
**Place of residence**				
Urban	Reference		Reference	
Rural	0.42 (0.23, 0.74)	0.003	1.09 (0.66, 1.80)	0.742

Abbreviations: *AOR* Adjusted Odds Ratio; *CI* Confidence Interval

In the subgroup of women who have ever been tested for HIV, marital status, education, and region were significantly associated with comprehensive HIV knowledge. Compared to women who have never been married, the odds of having a comprehensive HIV knowledge were lower in women who were married or living with a partner (OR 0.62, 95% CI: 0.46–0.83; *P = 0.002*). Compared to women with no education, the odds of having a comprehensive HIV knowledge were higher in women who had primary (OR 1.87, 95% CI: 1.07–3.27; *P = 0.029*) and secondary or above education (OR 2.87, 95% CI: 1.62–5.07; *P < 0.001*). Compared to women from the Addis Ababa region, the odds of having a comprehensive HIV knowledge were higher for women from the Amhara (OR 1.95, 95% CI: 1.23–3.08; *P = 0.004*) and Tigray (OR 1.65, 95% CI: 1.06–2.55; *P = 0.026*) regions. However, the odds of having a comprehensive HIV knowledge were lower for women from Dire Dawa region (OR 0.57, 95% CI: 0.34–0.96*; P = 0.034*). Finally, the odds of having a comprehensive HIV knowledge were higher in women who reported having access to the internet compared to women who had no internet access (OR 0.57, 95% CI: 0.34–0.95; *P = 0.033*) (Table 3).

## Discussion

In a large, nationally representative sample of Ethiopian youth, HIV testing moderated the association between place of residence and comprehensive HIV knowledge. We found a negative association between rural residence and comprehensive HIV knowledge only in the subgroup of women who have never been tested for HIV. The observed association was independent of potential confounders. Our results contribute to the existing literature by reporting, a negative association between rural residence and comprehensive HIV/AIDS knowledge in young women who have never been tested for HIV.

Findings from the current study suggest that comprehensive HIV knowledge among 15–24 years age women in Ethiopia is low, particularly among rural women. These findings are consistent with results from other studies in Ethiopia [21] but markedly lower than estimates from similar studies in Ghana (59%), and Uganda (38%*)* [29, 30]. This might be due to variation in socio-demographic characteristics of the participants and variation in level of HIV/AIDS related information exposure among countries. The proportion of comprehensive HIV knowledge among young women age 15–24 years has increased slightly from the year 2005 (21%) to 2011 (24%) and 2016 (24.8%). The prevalence of comprehensive knowledge in this analysis is identical to that of a 2011 study of high school students in eastern Ethiopia, Ethiopian adolescents in 2011 [21] and far lower than similar studies in Ghana (59%), and Uganda (38%*)* [31, 32]. This might be due to variation in socio-demographic characteristics of the respondents and variation in level of HIV related information exposure among countries.

The current study revealed that, among women who had never been tested for HIV, rural residents had lower odds of having a comprehensive HIV knowledge as compared to urban dwellers. Studies elsewhere have reported disparities in comprehensive HIV/AIDS knowledge by place of residence [29, 30, 33, 34]. Most of these studies reported lower rates of comprehensive HIV/AIDS knowledge among rural residents. However, none of these studies stratified their analysis by HIV testing. Plausible explanations for why rural residents may be have lower rates of comprehensive HIV/AIDS knowledge include, lower education, poverty, and limited or no access to HIV testing services [21, 35, 36]. The lower odds of comprehensive HIV/AIDS knowledge among women who had never been tested for HIV observed in the current study can be attributed to lack of access to HIV/AIDS resources and services including HIV testing in these settings. Previous studies have shown that testing can improve knowledge about HIV/AIDS [37–40]. Rural women who have never been tested for HIV are less likely to be exposed to the HIV counseling services that are often packaged with HIV testing services.

In the present study, young women residing in poorest households were less likely to have comprehensive HIV knowledge as compared to the richest counterparts. This finding is consistent with the report from Uganda [31]. This could be due to the fact that rich women can easily afford and access information from media and services [41, 42]. However, this result was contradicted with the finding reported from Ghana and Burkina Faso [32, 43]. This discrepancy might be due to differences in other country-specific socioeconomic characteristics associated with accessing sources of HIV related information. For example media exposure in Ghana (86.4%) is much higher than Ethiopia (31.9%). Similarly, a slightly higher proportion of participants from Ethiopia were rural (74.7%) compared the study from Burkina Faso (68.2%).

In our finding, respondents who had no information exposure at least through newspaper, radio, and television had lower odds of comprehensive HIV/AIDS knowledge compared to those with information exposure at least from one source. This suggests that media serves as a valuable source of information about HIV [44, 45]. In addition, respondents who had ever had HIV test had significantly higher odds of comprehensive HIV knowledge as compared to those who had never tested. This is in agreement with a study conducted in Kenya which revealed that those women who had ever tested for HIV were 1.6 times as likely to have comprehensive HIV knowledge compared to those who were not tested [34]. Similar results were reported for Burkina Faso and Sierra Leone [43, 46]. This could be due to the fact that, during HIV testing the individual might get counseling regarding HIV and related information [47]. However, in this study the proportion of comprehensive HIV knowledge among those who had ever been tested for HIV was low even though it is higher than those who had never been tested for HIV. This may indicate that respondents who had ever been tested for HIV were not successfully counseled about HIV or the counseling were not effective in addition to knowing their status. This implies the need to enhance organized counseling of youth during testing of their HIV status. Consequently, sustainable and reliable interventions that are sensitive to traditional, socio-cultural, and economic factors should be developed and implemented.

The positive association between increasing level of education and comprehensive HIV/AIDS knowledge have previously been documented [29, 34, 48, 49]. Our results add to this evidence by indicating that having some education is positively associated with comprehensive HIV knowledge, regardless of HIV testing. A possible explanation is that education empowers women and given them the opportunity to understand and process HIV/AIDS-related messages than less educated counterparts [41, 42].

The lower odds of having a comprehensive HIV knowledge in married or cohabiting women compared to women who have never been married observed in the subgroup of women who had been tested for HIV sheds light on the moderating role of marital status on comprehensive HIV knowledge. This finding is consistent with existing literature [34, 50]. One plausible explanation is that married or cohabiting women are more likely to have a loss of interest and may assume that they will benefit from their husband’s knowledge of HIV/AIDS [34, 51]. Although factors contributing to the geographic variations in the comprehensive HIV/AIDS knowledge are beyond the scope of this research, the regional differential in comprehensive HIV/AIDS knowledge observed in the current study could be attributed to differences in the HIV/AIDS related information, programs and services [52, 53]. Further research is needed to fully evaluate the content and nature of HIV/AIDS related programs and services.

### Strength and limitation of the study

There are a few limitations to consider in the present study. First, due to the cross-sectional design, the observed results cannot be interpreted as causal. Second, the self-reported measures of both comprehensive HIV knowledge and HIV testing are subject to measurement, self-report, social desirability, and recall biases. Third, data on women’s exposure to information on HIV prevention and transmission, HIV status that could influence women’s comprehensive HIV knowledge were not captured. The main strength is the use of a large sample that is representative young women aged 15–24 years in Ethiopia. Additionally, the DHS program standardized data collection methods with significant resources devoted to collecting accurate data. The availability of several variables in the DHS data allowed us to control for potential confounders in the multivariable-adjusted regression models.

## Conclusions

The findings of this study indicate that comprehensive HIV knowledge among young Ethiopian women is low. The current finding is unique because a negative association between place of residence and comprehensive HIV knowledge was present only in women who had never been tested for HIV. Such low level of comprehensive HIV knowledge among young women in Ethiopia suggests the need to scale-up ongoing HIV prevention efforts among rural and impoverished population. The current findings suggest that the development, implementation of any HIV prevention, testing, and care should tailored based on local context. Ongoing HIV related awareness and prevention efforts should target rural, poor and uneducated women who have limited access to HIV testing.

## Data Availability

Data is available and it can be accessed from the corresponding author with a reasonable inquiry.

## Abbreviations

ART: Antiretroviral therapy
CI: Confidence interval
DHS: Demographic and health surveys
STD: Sexual transmitted disease
HIV: Human immunodeficiency virus
UNAIDS: United Nation program on HIV/AIDS
MTCT: Mother to child transmission
OR: Odds ratio
UNICEF: The United Nations children’s fund
WHO: World health organization
